# Selection and validation of appropriate reference genes for RT-qPCR analysis of flowering stages and different genotypes of *Iris germanica* L

**DOI:** 10.1038/s41598-021-89100-y

**Published:** 2021-05-10

**Authors:** Yinjie Wang, Yongxia Zhang, Qingquan Liu, Haiying Tong, Ting Zhang, Chunsun Gu, Liangqin Liu, Suzhen Huang, Haiyan Yuan

**Affiliations:** Institute of Botany, Jiangsu Province and Chinese Academy of Sciences, Nanjing, 210014 China

**Keywords:** Molecular biology, Plant sciences

## Abstract

*Iris germanica* L. is a perennial herbaceous plant that has been widely cultivated worldwide and is popular for its elegant and vibrantly colorful flowers. Selection of appropriate reference genes is the prerequisite for accurate normalization of target gene expression by quantitative real-time PCR. However, to date, the most suitable reference genes for flowering stages have not been elucidated in *I. germanica*. In this study, eight candidate reference genes were examined for the normalization of RT-qPCR in three *I. germanica* cultivars, and their stability were evaluated by four different algorithms (GeNorm, NormFinder, BestKeeper, and Ref-finder). The results revealed that *IgUBC* and *IgGAPDH* were the most stable reference genes in ‘00246’ and ‘Elizabeth’, and *IgTUB* and *IgUBC* showed stable expression in ‘2010200’. *IgUBC* and *IgGAPDH* were the most stable in all samples, while *IgUBQ* showed the least stability. Finally, to validate the reliability of the selected reference genes, the expression patterns of *IgFT* (*Flowering Locus T* gene) was analyzed and emphasized the importance of appropriate reference gene selection. This work presented the first systematic study of reference genes selection during flower bud development and provided guidance to research of the molecular mechanisms of flowering stages in *I. germanica*.

## Introduction

Quantitative real-time PCR (RT-qPCR) is a reliable and widely used technique to quantify target gene expression patterns in various fields of biological research, due to its high sensitivity, accuracy and reproducibility^1–3^. However, the accuracy of RT-qPCR is influenced by various factors, including the quantity of mRNA templates, enzymatic efficiency in cDNA synthesis and PCR amplification^4^. Thus, to avoid bias, it is necessary to select reliable reference genes that are steadily expressed under different experimental conditions before determining the expression pattern of a target gene by RT-qPCR.

Traditional reference genes, such as elongation factor 1 alpha (*EF1α*), glyceraldehyde-3-phosphate dehydrogenase (*GAPDH*), actin (*ACT*) and ubiquitin (*UBQ*), are mostly involved in intermediary metabolism or other basic cellular processes^5–7^, and are therefore commonly accepted for normalization without the need for any validation for their stability. However, numerous studies indicate that the expression levels of these genes vary considerably at different developmental stages or throughout the entire lifecycle of plants^8,9^. A systematic study for each tested species is recommended for identifying the best potential reference genes. Furthermore, several statistical algorithms, such as GeNorm^10^, NormFinder^11^ and BestKeeper^12^, have been developed for the evaluation of potential reference gene(s) in different experimental systems.

Flowers of higher plants are reproductive organs that are widely studied and are important ornamental characteristics of ornamental plants. A number of reference genes have been reported for different flowering stages of various ornamental plants species, such as chrysanthemum^13^, petunia^14^, azalea^15^ and tree peony^16^, but little information is available concerning reference genes for *Iris*. *Iris germanica* L., which is often referred to as Pogon iris, is one of the most important ornamental species in the *Iris* genus^17^, the posture of the flower is peculiar and the flowers are rich and have high ornamental and economic value. However, the spring-time flowering habit and short duration of flowering of *I. germanica* hinder its year-round supply and economic benefits. To enable additional detailed and in-depth studies of the expression level of key genes involved in flowering, it is necessary to identify the stability of candidate reference genes at various flowering stages in *I. germanica*. Recently, studies have been shown that *ACT11* performed well in different tissues but poorly in different flower development stages in *I. germanica* cultivar ‘Huangjinjia’. Furthermore, *TUA* performed best in different flower development stages but was the worst in different tissues^18^. Nevertheless, limited information is available concerning reference genes for flowering stages in *I. germanica*.

In the present study, the expression stability of eight candidate reference genes, *IgEF1α*, *IgGAPDH*, *IgACT6*, *IgUBQ*, *IgUBC* (ubiquitin-protein ligase), *IgEF1β* (elongation factor 1β), *IgPGK* (phosphoglycerate kinase), *IgTUB* (beta-tubulin), was validated by RT-qPCR during flower development in different cultivars of *I. germanica*. Four statistical algorithms, GeNorm^10^, BestKeeper^12^, NormFinder^11^, and Ref-finder (http://www.leonxie.com/referencegene.php) were used to evaluate the most suitable reference genes needed for normalization. To verify the usefulness of the selected reference genes, the relative expression levels of *IgFT*, a putative homolog of *Flowering Locus T* (*FT*) gene in *Arabidopsis*, was analyzed during flower bud development and in different genotypes of *I. germanica*. This is the first report about the selection of reference genes during flower bud development and will benefit future studies on gene expression of flowering stages in *I. germanica* and other related species.

## Results

### Assessment of primer specificity and amplification efficiency of reference genes

The gene names, primer sequences and amplicon length characteristics of the 8 reference genes are summarized in Table 1. The primer specificities were verified by agarose gel electrophoresis, only a specific product of the expected size was observed, and no primer dimers were detected (Supplementary Fig. S1). In addition, only a single peak was found in the melting curves of the amplified products of all genes, indicating that no primer dimers were generated (Supplementary Fig. S2). The amplification efficiency (E) of each reference gene varied from 98.47% for *IgGAPDH* to 101.74% for *IgUBQ*, and the correlation coefficients (*R*^*2*^) ranged from 0.9984 to 0.9998 (Table 1). The amplicon size ranged from 107 for *IgACT6* to 295 bp for *IgUBQ*.

**Table 1:** Primer sequences and characteristics of PCR amplifications in *I. germanica*.

Gene	Description	Accession numbers	*Arabidopsis* ortholog	Primer sequence (forward/reverse)	Product size (bp)	Primers TM (℃)	Amplification efficiency (%)	R^2^
*IgEF1α*	Elongation factor 1 alpha	MN602628	At1G07940	5′-ACCATACCAGGCTTGATAACTCC-3′ 5′-AGACTGGTACAAGGGTCCTACTCTC-3	171	59.4/59.5	100.63%	0.9995
*IgGAPDH*	Glyceraldehyde-3-phosphate dehydrogenase	MN602629	At2G24270	5′-TTTGCTGACGACTCGGACAC-3′ 5′-CTTGGATTTGGTTGCTGCTAAT-3′	204	59.1/59.1	98.47%	0.9998
*IgACT6*	Actin6	MN602630	At2G31200	5′-TGCTGCTTTGATTGCGTGT-3′ 5′-AGCTCCATACAATCGACTCAGG-3′	107	58.2/58.6	98.74%	0.9985
*IgUBQ*	Ubiquitin	MN602631	At4G05320	5′-GATGGTCGCACACTTGCTGA-3′ 5′-GGAGCCTGAGAACAAGATGGA-3′	295	59.9/59.1	101.74%	0.9997
*IgUBC*	Ubiquitin-protein ligase	MN602632	At5G53300	5′-CCTCCCTTTCCAATCGCTAA-3′ 5′-AGGTGCTGCTGTCCATCTGTT-3′	162	59.5/59.5	101.67%	0.9997
*IgEF1β*	Elongation factor 1β	MN602633	At5G19510	5′-TTGGAGGAGACCGTTCGC-3′ 5′-TCATTGGCAGGCTCAACAGT-3′	173	58.8/58.7	100.64%	0.9995
*IgPGK*	Phosphoglycerate kinase	MN602634	At1G56190	5′-GTTGTGCCAGCGTCTGAAAT-3’ 5′-ACCTCGGCTACTCCCACTTT-3′	263	58.4/58.0	101.19%	0.9994
*IgTUB*	Beta-tubulin	MN602635	At1G64740	5′-GTTTGACTTCCAGTTTGGTTGTG-3′ 5′-GCAAAACAAACACCCGCTTA-3′	274	59.0/59.0	98.77%	0.9984
*IgF3H*	Flavanone 3-hydroxylase	MN602636	At3G51240	5′-GGTTCATTGTCTCCAGCCATC-3′ 5′-ATTGCTTCGGAGAGGACCC-3′	202	59.2/58.8	98.70%	0.9991

### Expression levels and profiles of reference genes

**Figure 1:**
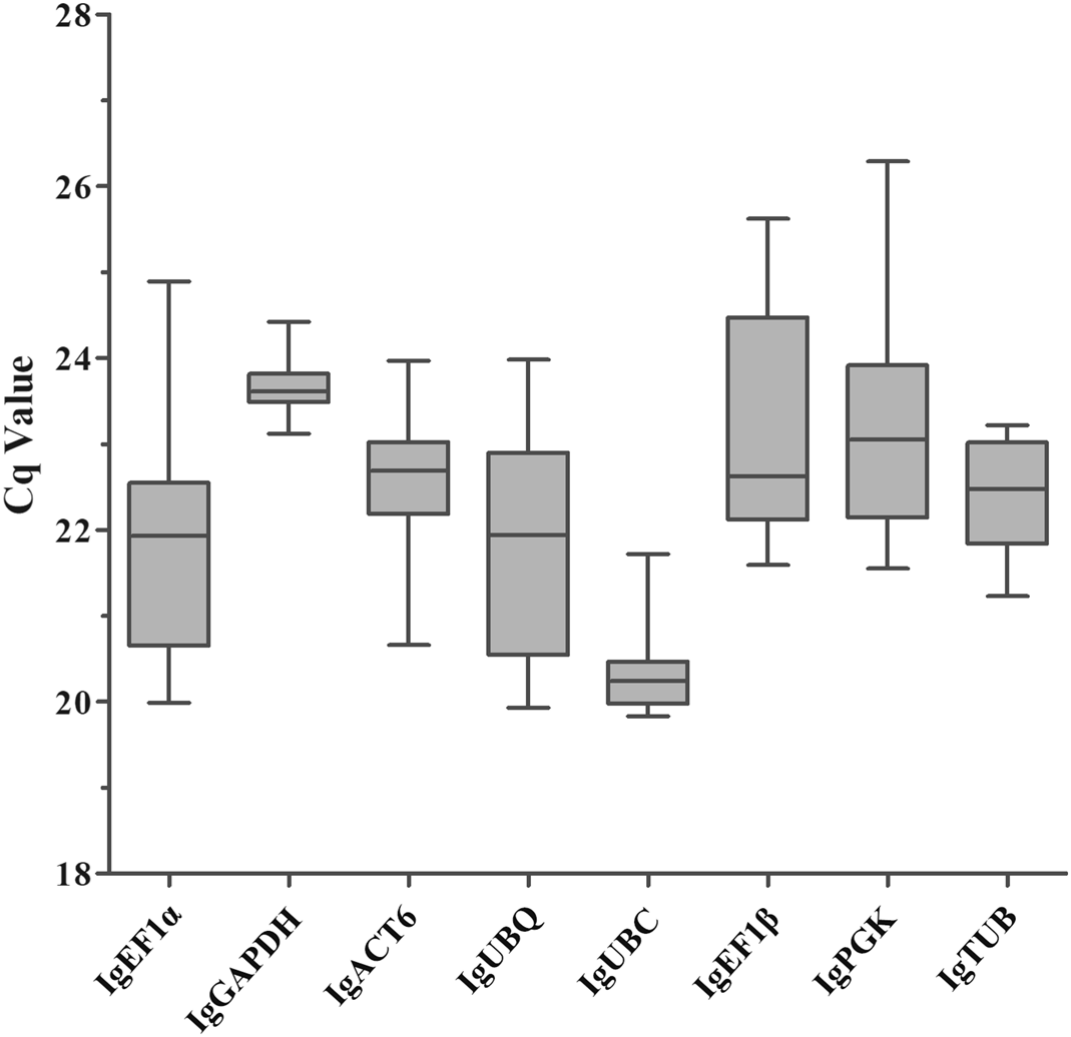
Cq values of the 8 candidate reference genes in all samples of *I. germanica.* Line across the box-plot of Cq value depicts median values. The outside box is determined by the 25th and 75th percentiles. The whiskers represent the maximum and minimum values.

The quantification cycle (Cq) values of eight reference genes were assayed by RT-qPCR analysis, with lower Cq values representing relatively higher mRNA transcript levels. Cq values for each of the 8 candidate reference genes in three different genotypes of *I. germanica* are listed in supplementary Table S1. The average Cq values of these reference genes ranged from 19.83 to 27.61 for the highest and lowest expression levels, respectively, across all samples (Fig. 1). In addition, the standard deviations (SD) of Cq values were used to determine reference gene stability levels. Genes with relatively high SD of Cq values showed more variable expression than did those with lower SD. *IgGAPDH* showed the least variation in gene expression (23.69 ± 0.36) and presented the lowest SD, while *IgEF1β* showed the most variable levels of expression (23.16 ± 1.38).

### Expression stability of reference genes

To further select the most appropriate reference gene for RT-qPCR-based analysis in the investigation of flower development across three different *I. germanica* cultivars, four software programs, GeNorm, NormFinder, BestKeeper and RefFinder were used to analyze the expression stability of each reference gene.

#### GeNorm analysis

**Figure 2:**
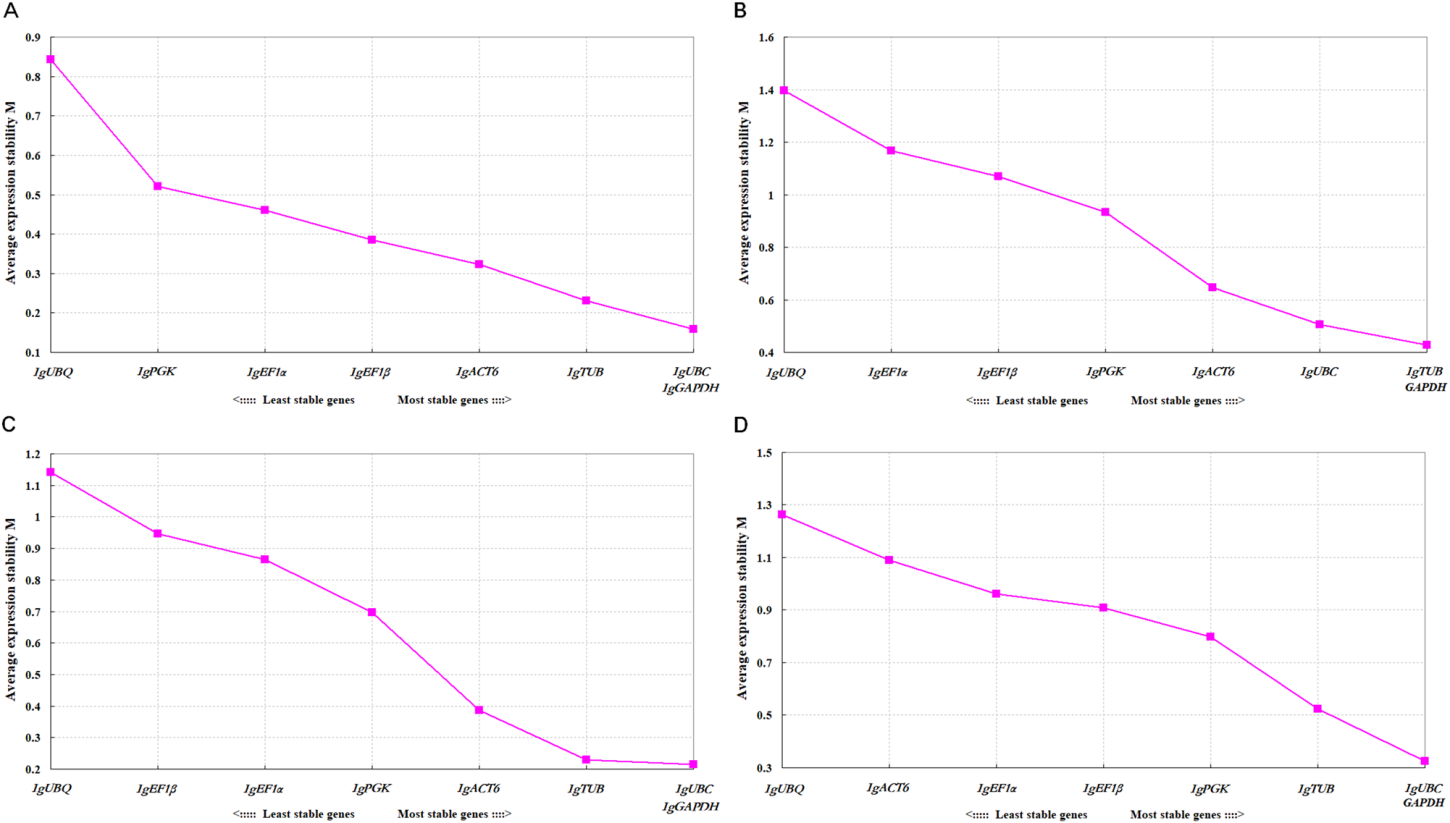
Average expression stability values (M) of 8 candidate reference genes predicted by GeNorm software. The most stable genes are on the right, while the least stable genes are on the left. (**A**) ‘00246’, (**B**) ‘2010200’, (**C**) ‘Elizabeth’, and (**D**) all samples.

GeNorm program was used to evaluate the expression stability of the 8 candidate reference genes by calculating average expression stability (*M*) values based on the average pairwise variation among all the tested genes. According to GeNorm algorithm, stably expressed genes had *M* values below 1.5, and a relatively low *M* value indicates a relatively stable expression^10^. In this study, all of the tested genes showed high expression stability, with *M*-values of < 1.5, indicating that they all conformed to basic requirements for function as reference genes. *IgUBC* and *IgGAPDH* were the most stable reference genes in both ‘00246’ and ‘Elizabeth’, while *IgTUB* and *IgGAPDH* were identified as the most stable in ‘2010200’. In terms of the total samples tested, *IgUBC* and *IgGAPDH* were recommended as the most stable reference genes. In contrast, *IgUBQ* with the highest *M* value was identified as the least stable reference gene in all of the samples (Fig. 2).

**Figure 3:**
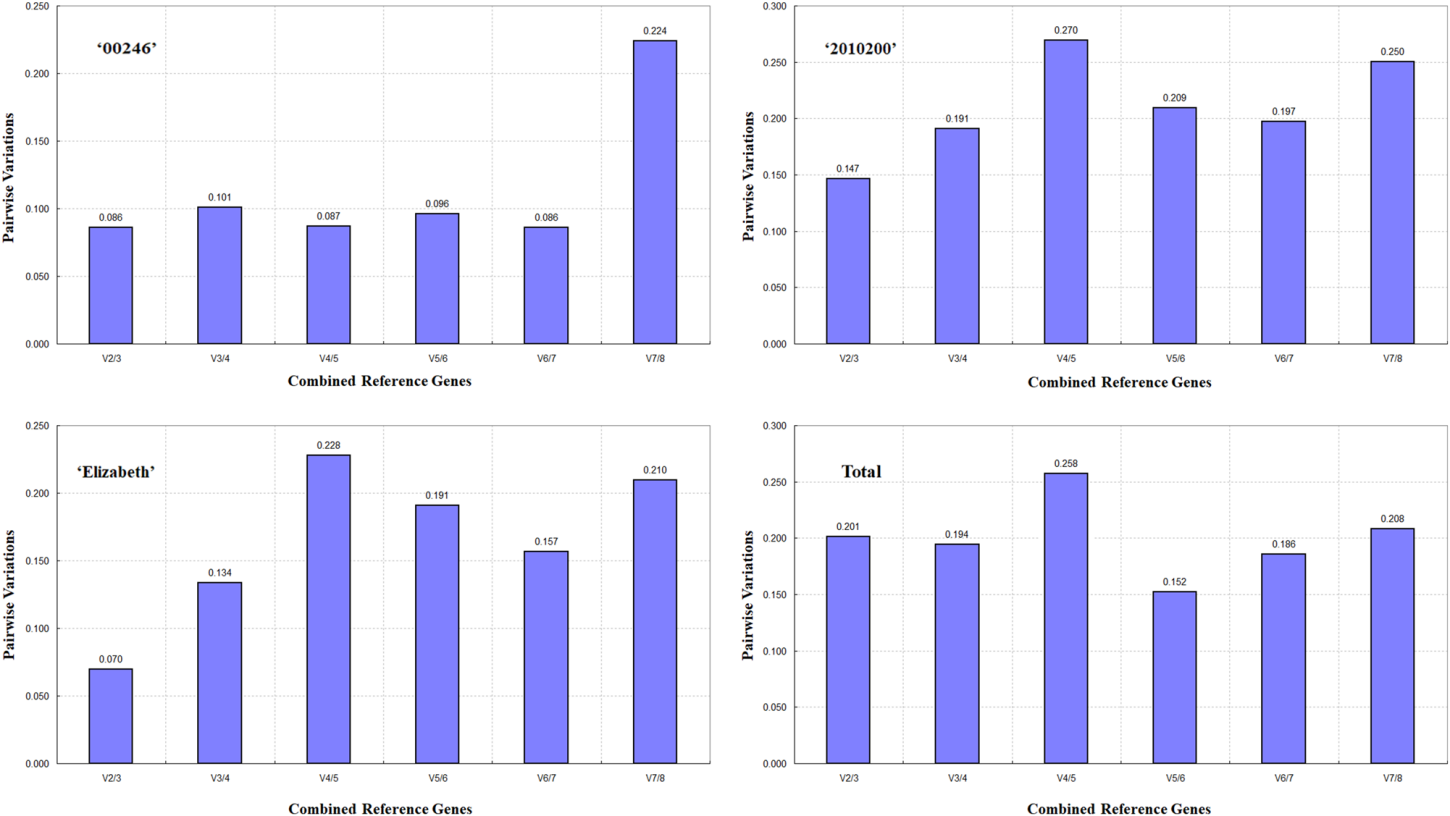
Pairwise variation (V) of 8 candidate reference genes were calculated by GeNorm. Vn/Vn + 1 value were used to determine the optimal number of reference genes.

The optimal number of reference genes was also measured by determining the pairwise variation between sequentially ranked genes (Vn/Vn + 1) based on the GeNorm algorithm (Fig. 3). Generally speaking, a cutoff of 0.15 (Vn value) has been recommended as the threshold to determine the optimal number of reference genes^10^. Our results reveal that the V2/3 values of the ‘00246’, ‘2010200’ and ‘Elizabeth’ samples were lower than 0.15 (Fig. 3), suggesting that two reference genes were sufficient for accurate normalization. However, the value of 0.15 should not be a fixed threshold, and higher cutoff values of Vn/n + 1 have been shown in several reports^19,20^. Our data showed small variation between V2/3 and V3/4 across all the samples, suggesting that two reference genes were sufficient for normalization (Fig. 3), which was similar to results in bermudagrass^21^ and Kentucky bluegrass^22^.

#### NormFinder analysis

NormFinder is used to determine the stability value of reference genes, based on inter- and intragroup variance in different sample groups^11^. The stability value is then calculated, with a relatively low stability value meaning that the gene is relatively stable. Reference gene stability values were calculated by NormFinder, as shown in Table 2. *IgUBC* and *IgGAPDH* were the two most stable genes among the total group, while *IgUBQ* was the least stable. The top two most stably expressed genes were *IgUBC* and *IgGAPDH* in both ‘00246’ and ‘2010200’, and *IgUBC* and *IgTUB* in ‘Elizabeth’. The ranking order generated by this method was slightly different from that of GeNorm.

**Table 2:** Expression stability analysis of 8 candidate reference genes calculated using NormFinder software.

Rank	‘00246’		‘2010200’		‘Elizabeth’		Total	
	Gene	Stability	Gene	Stability	Gene	Stability	Gene	Stability
1	*IgGAPDH*	0.055	*IgUBC*	0.187	*IgUBC*	0.074	*IgUBC*	0.112
2	*IgUBC*	0.055	*IgGAPDH*	0.343	*IgTUB*	0.172	*IgGAPDH*	0.207
3	*IgTUB*	0.078	*IgTUB*	0.363	*IgGAPDH*	0.200	*IgTUB*	0.442
4	*IgACT6*	0.268	*IgPGK*	0.584	*IgACT6*	0.534	*IgPGK*	0.586
5	*IgEF1β*	0.308	*IgEF1β*	0.811	*IgPGK*	0.617	*IgEF1ɑ*	0.715
6	*IgEF1ɑ*	0.512	*IgACT6*	0.814	*IgEF1ɑ*	0.723	*IgEF1β*	0.772
7	*IgPGK*	0.537	*IgEF1ɑ*	1.044	*IgEF1β*	0.876	*IgACT6*	0.820
8	*IgUBQ*	1.179	*IgUBQ*	1.338	*IgUBQ*	1.101	*IgUBQ*	1.093

#### BestKeeper analysis

BestKeeper evaluates the stability of reference genes based on the standard deviation (SD) and coefficients of variation (CV) of Cq values, with relatively low SD and CV representing relatively high stability^12^. The results of BestKeeper analysis are listed in Table 3. *IgGAPDH* and *IgUBC* were recommended as the most stable genes in ‘00246’, ‘Elizabeth’ and across all the samples, which was similar to the results from the GeNorm and NormFinder analysis. In ‘2010200’ samples, *IgTUB* and *IgACT6* were detected as the most stable genes via BestKeeper analysis, whereas *IgACT6* was ranked fourth by GeNorm and sixth by NormFinder.

**Table 3:** Expression stability analysis of 8 candidate reference genes calculated using BestKeeper software.

Rank	‘00246’		‘2010200’		‘Elizabeth’		Total	
	Gene	CV ± SD	Gene	CV ± SD	Gene	CV ± SD	Gene	CV ± SD
1	*IgGAPDH*	0.33 ± 0.08	*IgTUB*	0.87 ± 0.20	*IgGAPDH*	0.66 ± 0.16	*IgGAPDH*	0.89 ± 0.21
2	*IgUBC*	0.62 ± 0.12	*IgACT6*	1.58 ± 0.36	*IgUBC*	0.92 ± 0.19	*IgUBC*	1.39 ± 0.28
3	*IgTUB*	1.25 ± 0.27	*IgGAPDH*	1.86 ± 0.45	*IgTUB*	2.96 ± 0.66	*IgTUB*	2.91 ± 0.65
4	*IgEF1β*	1.74 ± 0.39	*IgUBC*	2.58 ± 0.53	*IgEF1ɑ*	3.52 ± 0.76	*IgACT6*	2.98 ± 0.68
5	*IgACT6*	2.00 ± 0.47	*IgPGK*	4.63 ± 0.97	*IgPGK*	3.67 ± 0.84	*IgPGK*	3.98 ± 0.92
6	*IgEF1ɑ*	2.62 ± 0.57	*IgEF1β*	4.70 ± 1.06	*IgACT6*	3.71 ± 0.84	*IgEF1ɑ*	4.07 ± 0.89
7	*IgPGK*	2.86 ± 0.64	*IgEF1ɑ*	5.77 ± 1.34	*IgEF1β*	4.26 ± 0.98	*IgEF1β*	4.38 ± 1.00
8	*IgUBQ*	5.22 ± 1.12	*IgUBQ*	6.92 ± 1.42	*IgUBQ*	4.46 ± 0.95	*IgUBQ*	5.23 ± 1.14

#### RefFinder analysis

RefFinder (http://www.leonxie.com/referencegene.php) was used to obtain the comprehensive rankings of reference genes by integrating three common analysis programs: GeNorm, NormFinder and BestKeeper^23^. The final comprehensive rankings of the three algorithms were integrated by RefFinder and the results are shown in Table 4. Across all the samples, the ranking order was (from the most stable to the least stable) as follows: *IgUBC* > *IgGAPDH* > *IgTUB* > *IgPGK* > *IgEF1ɑ* > *IgACT6* > *IgEF1β* > *IgUBQ*. This order is similar to the results of the GeNorm and NormFinder analysis. *IgGAPDH* and *IgUBC* were ranked as the most stable genes in ‘00246’ and ‘Elizabeth’, and *IgTUB* and *IgUBC* were the most stable genes in ‘2010200’. On the other hand, *IgUBQ* was the most unstable gene in all the samples, ‘00246’ and ‘2010200’, and *IgEF1β* was the least stable gene in ‘Elizabeth’. In the all samples, *IgUBC* and *IgGAPDH* were purported to be the most stable reference genes, while *IgUBQ* showed the least stability.

**Table 4:** Expression stability analysis of 8 candidate reference genes calculated using RefFinder software.

Method	1	2	3	4	5	6	7	8
**Total**								
Comprehensive ranking	*UBC*	*GAPDH*	*TUB*	*PGK*	*EF1ɑ*	*ACT6*	*EF1β*	*UBQ*
GeNorm	*UBC/GAPDH*		*TUB*	*PGK*	*EF1β*	*EF1ɑ*	*ACT6*	*UBQ*
NormFinder	*UBC*	*GAPDH*	*TUB*	*PGK*	*EF1ɑ*	*EF1β*	*ACT6*	*UBQ*
Bestkeeper	*GAPDH*	*UBC*	*TUB*	*ACT6*	*PGK*	*EF1ɑ*	*UBQ*	*EF1β*
**‘00246’**								
Comprehensive ranking	*GAPDH*	*UBC*	*TUB*	*ACT6*	*EF1β*	*EF1ɑ*	*PGK*	*UBQ*
GeNorm	*UBC/GAPDH*		*TUB*	*ACT6*	*EF1β*	*EF1ɑ*	*PGK*	*UBQ*
NormFinder	*GAPDH*	*UBC*	*TUB*	*ACT6*	*EF1β*	*EF1ɑ*	*PGK*	*UBQ*
Bestkeeper	*GAPDH*	*UBC*	*TUB*	*EF1β*	*ACT6*	*EF1ɑ*	*PGK*	*UBQ*
**‘2010200’**								
Comprehensive ranking	*TUB*	*UBC*	*GAPDH*	*ACT6*	*PGK*	*EF1β*	*EF1ɑ*	*UBQ*
GeNorm	*TUB/GAPDH*		*UBC*	*PGK*	*EF1β*	*EF1ɑ*	*ACT6*	*UBQ*
NormFinder	*UBC*	*GAPDH*	*TUB*	*PGK*	*EF1β*	*ACT6*	*EF1ɑ*	*UBQ*
Bestkeeper	*TUB*	*ACT6*	*GAPDH*	*UBC*	*UBQ*	*PGK*	*EF1β*	*EF1ɑ*
**‘Elizabeth’**								
Comprehensive ranking	*UBC*	*GAPDH*	*TUB*	*ACT6*	*PGK*	*EF1ɑ*	*UBQ*	*EF1β*
GeNorm	*UBC/GAPDH*		*TUB*	*ACT6*	*PGK*	*EF1ɑ*	*EF1β*	*UBQ*
NormFinder	*UBC*	*TUB*	*GAPDH*	*ACT6*	*PGK*	*EF1ɑ*	*EF1β*	*UBQ*
Bestkeeper	*GAPDH*	*UBC*	*TUB*	*ACT6*	*UBQ*	*EF1ɑ*	*PGK*	*EF1β*

### Validation of the selected reference genes

**Figure 4:**
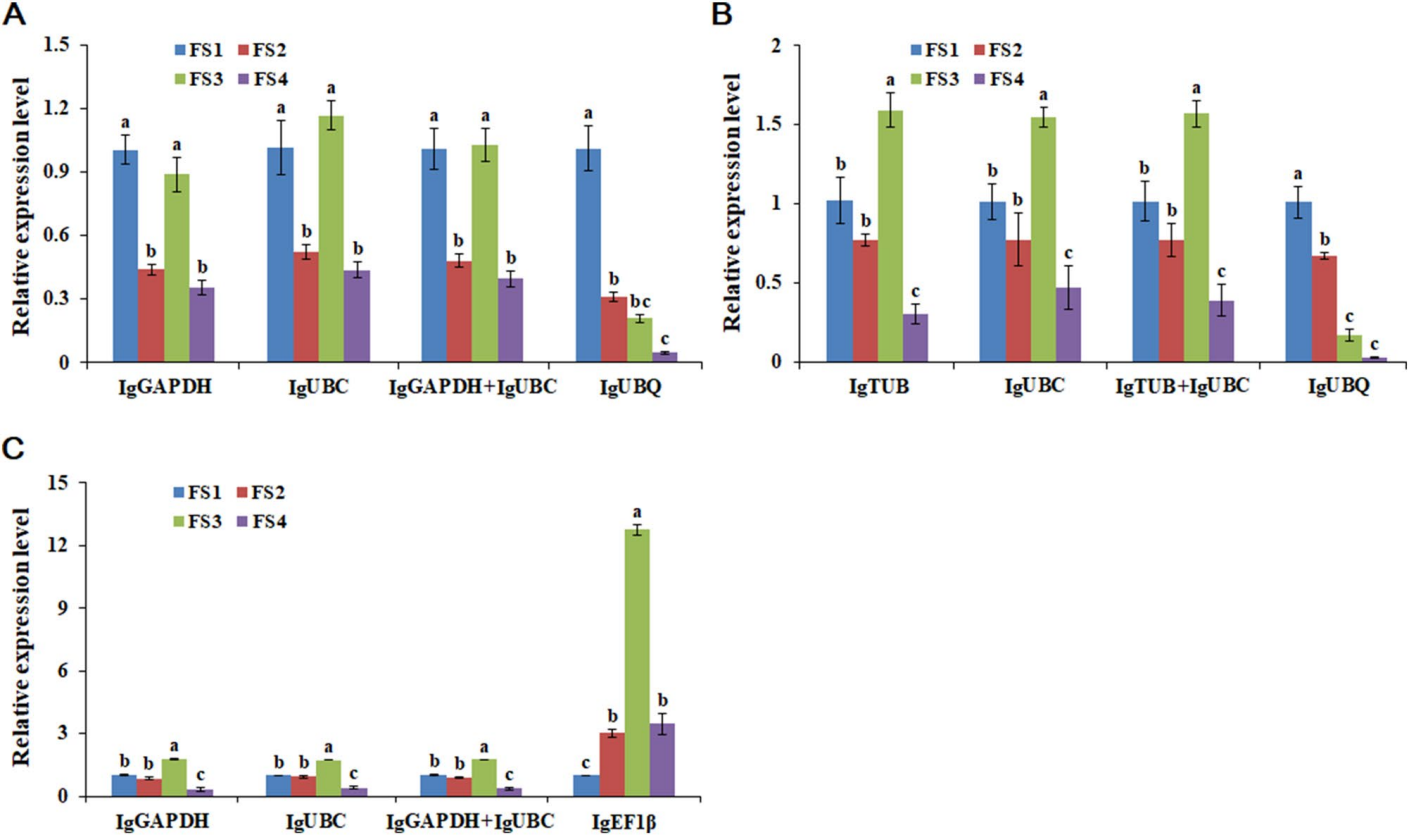
Relative quantification of *IgFT* gene expression in different cultivars of *I. germanica* using validated reference genes for normalization. (**A**) The expression level of *IgFT* in ‘00246’. (**B**) The expression level of *IgFT* in ‘2010200’. (**C**) The expression level of *IgFT* in ‘Elizabeth’. The bars represent standard errors. The relative expression level is reported as the mean of three biological replicates (n = 3), with bars indicating the standard deviation. Different letters indicate a statistically significant difference in each condition (*P* < 0.05, student’s t-test).

To validate the reliability of the reference genes, the relative expression patterns of *IgFT* were examined using different combinations of reference genes in the three cultivars. The two most stable reference genes (*IgGAPDH* and *IgUBC* for ‘00246’ and ‘Elizabeth’, *IgTUB* and *IgUBC* for ‘2010200’) and the least stable reference genes (*IgUBQ* for ‘00246’ and ‘2010200’, *IgEF1β* for ‘Elizabeth’) selected from the analyses described above were used either alone or in combination for RT-qPCR analyses. As shown in Fig. 4, although the overall relative expression patterns of *IgFT* showed similar trends, differences were found when the data were normalized to those of the different reference genes. When the least stable gene *IgUBQ* was used as the reference gene, the normalized expression levels of *IgFT* in ‘00246’ and ‘2010200’ significantly decreased compared with those normalized using *IgGAPDH* or *IgUBC* alone, the combination of *IgGAPDH* + *IgUBC* (for ‘00246’), *IgTUB* or *IgUBC* alone or the combination of *IgTUB* + *IgUBC* (for ‘2010200’) (Fig. 4A, B). However, when the least stable gene *IgEF1β* was used for normalization, the expression level of *IgFT* dramatically increased compared to that of *IgGAPDH*, *IgUBC*, or the combination of *IgGAPDH* + *IgUBC* in ‘Elizabeth’ (Fig. 4C). The combination of *IgGAPDH*/*IgUBC* was recommended as the optimum pair of reference genes for ‘00246’ and ‘Elizabeth’, and *IgTUB*/*IgUBC* was the best suited pair of reference genes for accurate normalization in ‘2010200’. *IgUBC* was the most suitable reference gene for three different *I. germanica* cultivars.

## Discussion

Gene expression analyses are extremely important for revealing the molecular mechanisms that regulate important plant traits^24,25^. RT-qPCR has become the most powerful technique for quantification studies at the mRNA transcript level^13^. Selecting the appropriate reference genes is a necessary prerequisite for reliable RT-qPCR-based analysis. Ideally, an accurate reference gene should display stable expression in different tissues, in different organs, at different developmental stages and across different treatments^26^. In this study, we performed a systematic evaluation of 8 reference genes at different flowering stages and different genotypes of *I. germanica* cultivars. This study is the first attempt to identify the reference genes suitable for RT-qPCR normalization in flowering stages of *I. germanica*. The expression stability of various reference genes differed among the cultivars. Similar results were reported in tree peony, *Panax ginseng* and strawberry^16,27,28^. Different genetic backgrounds and biological processes between cultivars may have important effects on the expression stability of reference genes. Indeed, the selection and validation of reliable reference genes for quantitative analysis of gene expression analysis were necessary for the different cultivars.

Three programs GeNorm, NormFinder and BestKeeper, which are based on different algorithms and analytical procedures, are widely used to select the most reliable reference genes by researchers^29,30^. In this study, we found discrepancies in the reference gene stability rankings and validation data generated by the three different algorithms above. The rankings created by GeNorm and NormFinder were similar, but they showed quite distinct differences from the ranking obtained by BestKeeper. For instance, *IgACT6* was ranked among the top two stable genes by BestKeeper in ‘2010200’ but was ranked in the middle or bottom portion by GeNorm and NormFinder. Moreover, across all the samples, *IgACT6* was ranked among the top four stable genes by BestKeeper, whereas it was ranked seventh by GeNorm and NormFinder. These results are similar to those of many previous studies, possibly due to the different principles among the algorithms^13,31^. RefFinder, a comprehensive statistical program that integrates data from GeNorm, NormFinder, and BestKeeper, is used to evaluate the overall stability of reference gene expression^32^ Based on the comprehensive analysis by Ref-finder, *IgGAPDH* and *IgUBC* for ‘00246’ and ‘Elizabeth’ and *IgTUB* and *IgUBC* for ‘2010200’ were identified as the most stable reference genes for RT-qPCR of target gene expression studies. These results suggest that all 8 reference genes exhibited differential stability among the three cultivars.

In this study, we evaluated 8 genes that have been widely used as candidate reference genes in many species. The results indicate that it is better to select different reference genes according to different biological samples. Based on the results of our study involving different flower developmental stages, *IgUBC*, *IgGAPDH* and *IgTUB* were good candidates for normalization in all of the samples. Similar studies have also been conducted in other species, such as Rhododendrons^33^, Chinese cabbage^34^, *Chrysanthemum lavandulifolium*^35^ and *Silybum marianum*^36^. Moreover, *UBQ* was determined to be one of the most stable reference genes under NaCl and Pb stress in *Iris. lactea var. chinensis*^37^, but this gene was ranked as the least stable reference genes in both ‘00246’ and ‘2010200’ in our study, which was similar to the findings in flower buds of *Iris bulleyana*^38^ and *Rhizophora apiculate*^39^. *EF1α* and *EF1β* were determined to be the best suitable reference genes for all samples of various tissues in soybean^40^. In our study, these two genes ranked very low in all the samples, and *IgEF1β* exhibited the most unstable expression values in ‘Elizabeth’, which was similar to results in Moso bamboo^41^. *ACT6* and *PGK* were determined to be the most stable reference genes for proper normalization in flower buds of *Iris bulleyana*^38^ and Chrysanthemum across ploidy levels^42^ and meiosis and somatic tissues of wheat^43^, while these two genes were found to be not well suited in our study, similar to reports in *I. lactea var. chinensis*^37^. *TUB*, a member of the Tubulin gene family, has also been widely used as a reliable reference gene in *Primula forbesii*^44^ and peach^45^. Similarly, in our study, *IgTUB* was determined as the most stable reference gene in the flowers of ‘2010200’. However, this gene ranked very low under all the tested conditions in *Iris bulleyana*^38^. *GAPDH* has been reported as the most stable reference gene under PEG and cold stress in *I. lactea var. chinensis*^37^, but it showed unstable expression under various environmental conditions in garlic plants^46^ and under PEG and NaCl treatments in *Glehnia littoralis*^47^. In our study, *IgGAPDH* was the most stable reference gene across all flower developmental stages in ‘00246’. In addition, *IgUBC* was ranked first in ‘Elizabeth’ and was also the most stable reference gene in all the samples, which was similar to the findings in all samples of *I. lactea var. chinensis*^48^. These results highlight the fact that the choice of reference genes for normalization should be specific. Even though the samples belong to the same type and are from the same species (but belong to different lines), they may have different sets of reference genes. Therefore, it is necessary to select and verify reliable reference genes for quantitative gene expression analysis, whether for different species or for different cultivars.

To illustrate the reliability of the reference genes, the expression levels of the *IgFT* gene were normalized by using the most stable or least stable reference genes. The results showed that the relative expression level of *IgFT* exhibited a clear pattern in all three cultivars when the stable reference genes *IgGAPDH*, *IgUBC*, and *IgTUB* or a combination of them were used (Fig. 4). The relative transcript abundance presented conflicting results when the least stable genes, *IgUBQ* or *IgEF1β* were used. Therefore, the selection of suitable internal control genes is critically important for normalization of target gene expression data by RT-qPCR.

In summary, the current study provides the first comprehensive analysis of the selection of stable reference genes as internal controls for RT-qPCR-based analysis of target gene expression in different flowering stages and different genotypes of *I. germanica* cultivars. *IgGAPDH* combined with *IgUBC* was recommended as the optimal reference gene in ‘00246’ and ‘Elizabeth’, while *IgUBC*/*IgTUB* was identified as the best combination for ‘2010200’. This research is the first report on the validation of candidate reference genes across flower developmental stages of three different *I. germanica* cultivars, which will provide basic data for research on the molecular mechanism underlying flower development in this species, and lays a foundation for similar studies in other related species.

## Materials and methods

### Plant material

*Iris germanica* L. materials were introduced from Xi'an, Beach, Xi'an, Shanxi Province (CHN, latitude 34° 15′ N, longitude 108° 56′ E) under the permission of competent authority and cultivated in the *Iris* Resource Collection Garden of Institute of Botany, Nanjing Sun Yat-Sen Memorial Botanical Garden (CHN, latitude 32° 01′ N, longitude 118° 48′ E). All experiments are carried out with relevant institutional, national, and international guidelines and legislation. Three different genotypes of *I. germanica* cultivars including ‘00246’ (an early-flowering cultivar), ‘2010200’ (an intermediate-flowering cultivar), and ‘Elizabeth’ (a late-flowering cultivar), were used as plant materials; these cultivars can be used to study the flowering gene expression of *I. germanica* in different flowering stages. For each cultivar, flower buds samples were collected from plants into four flowering stages: in flowering stage 1 (FS1), the size of flower bud is less than 1.00 mm in length, in flowering stage 2 (FS2), the size of flower bud is between 1.00 to 2.00 mm, in flowering stage 3 (FS3), the size of flower bud is between 2.00 to 3.00 mm, in flowering stage 4 (FS4), the size of flower bud is between 4.00 to 5.00 mm. The traits of three different genotypes of *I. germanica* cultivars were shown in Table S2. The samples were immediately frozen in liquid nitrogen and stored at  − 80 ℃ until RNA extraction. The experiment included three biological replicates.

### RNA isolation and cDNA synthesis

Total RNA was extracted using RNA simple Total Kit (TaKaRa Dalian, China) according to the manufacturer’s instructions. Total RNA was pretreated with RNase-free DNase I (TaKaRa, Dalian, China) at 37 ℃ for 30 min to eliminate potential DNA contamination. The integrity of the total RNA was assessed by 1.5% (w/v) agarose gel electrophoresis and the concentration of the samples was determined by a NanoDrop ND-1000 spectrophotometer (NanoDrop Technologies, USA). Only RNA samples showing an A_260_/A_280_ ratio of 1.9–2.1 and A_260_/A_230_ ratio > 2.0 were used for subsequent analysis. For qPCR based analysis, first strand cDNA was synthesized using 1 μg total RNA in a volume of 20 μL with the PrimeScript RT Reagent Kit (TaKaRa, Dalian, China) according to the manufacturer’s instructions.

### Selection of reference genes and PCR primer design

Eight candidate reference genes, *IgEF1α*, *IgGAPDH*, *IgACT6*, *IgUBQ*, *IgUBC*, *IgEF1β*, *IgPGK* and *IgTUB* were selected from homologs of traditional housekeeping genes previously used for flower development^13,27^. The putative homologs of 8 reference genes were identified from the transcriptomic data sequences of *Iris lactea var. chinensis*^49^. All the candidate reference genes were cloned in Stage 4 samples and confirmed through sequencing. Primers were designed using Primer 5.0 software (Premier Biosoft International) with melting temperatures (TM) of 55–65 ℃, primer lengths of 18–25 bp and amplicon lengths of 107–295 bp (Table 1). The performance of the primers was tested by qPCR and the specificity of the primer amplicons was further verified by 2% (w/v) gel electrophoresis.

### Quantitative real-time PCR (RT-qPCR)

qPCR was performed using a Mastercycler ep realplex 2S device (Eppendorf, Germany) in conjunction with SYBR Premix Ex Taq II (TaKaRa, Dalian, China). Reactions were performed in a total volume of 20 μL containing 5 μL of diluted cDNA, 0.6 μL of each of forward and reverse primer (10 μM), 10 μL of 2 × SYBR Premix and 3.8 μL of ddH_2_O. The amplification program comprised an initial denaturation step (95℃ for 2 min), followed by 40 cycles of 95℃ for 5 s, 60℃ for 30 s, and 72 ℃ for 30 s, and a melting curve protocol (60–95 ℃ with a temperature increment of 0.5 ℃ s^−1^). Each RT-qPCR was performed for three biological and three technical replicates, and negative controls were included for each primer pair. Amplification efficiency (E) and correlation coefficient (*R*^*2*^) values were obtained from standard curves generated using a tenfold diluted cDNA series, the starting quantity of cDNA was 500 ng/μL^50^.

### Data analysis

The stability of the eight candidate reference genes was assessed using GeNorm^10^, NormFinder software^11^, BestKeeper^12^, and Ref-finder (http://www.leonxie.com/referencegene.php). For GeNorm and NormFinder analysis, quantification cycle (Cq) values were transformed into relative expression levels using the formula: 2^−∆Cq^, in which ∆Cq = each corresponding Cq value-the minimum Cq value^16^. The expression stability measurement (*M*) was determined by the GeNorm program based on the average pairwise variations (V) among all the other reference genes. NormFinder program estimates intra- and intergroup variations, and the lowest stability is ranked the highest. Calculations of the BestKeeper program are calculated based on the coefficients of variation (CV) and the standard deviations (SD) of the Cq values, and the lowest CV and SD were used as detection indexes for the most-stable reference genes. Ref-finder is an online tool that integrates the results of the currently available major computational programs, including GeNorm (*M* values), NormFinder (stability values), BestKeeper (CV and SD), and ∆Cq values.

### Validation of selected reference genes

To validate the influence of the choice of different reference genes on the final normalized outcome, the relative expression levels of *IgFT* which plays an important role in promoting flowering^51^ in three cultivars were analyzed using individual stably expressed or unstably expressed genes or a combination of stable reference genes, as determined by GeNorm^48^. The primers used for *IgFT* are presented in Table 1. The fold change of gene expression was calculated using the 2^−∆∆Ct^ method^24^.

## Conclusions

This research provided the first systematic analysis for the selection of stable reference genes as the internal control in RT-qPCR analysis in different flowering stages and different genotypes of *I. germanica* cultivars. Analysis using GeNorm, NormFinder, BestKeeper, and Ref-finder revealed that *IgUBC*, *IgGAPDH*, and *IgTUB* could be considered as appropriate reference genes for gene expression analysis in future molecular researches that aim to understand the mechanisms of flowering stages in *I. germanica*.

## Supplementary Information

Supplementary Information.
