# Supplementary material for: Selection and validation of appropriate reference genes for RT-qPCR analysis of flowering stages and different genotypes of Iris germanica L
Source: Sci Rep. 2021 May 10;11:9901. doi: 10.1038/s41598-021-89100-y (PMC8110784; doi:10.1038/s41598-021-89100-y)
Supplement: Supplementary file 1 — Supplementary Information. [file 41598_2021_89100_MOESM1_ESM.docx]

**Selection and validation of a****ppropriate reference genes for RT-qPCR analysis of flowering stages and** **different genotypes of *Iris germanica* L.**

**Authors:** Yinjie Wang, Yongxia Zhang, Qingquan Liu, Haiying Tong, Ting Zhang, Chunsun Gu, Liangqin Liu, Suzhen Huang and Haiyan Yuan*

**Address:** Institute of Botany, Jiangsu Province and Chinese Academy of Sciences, Nanjing 210014, China

***Corresponding author:**

Haiyan Yuan

Institute of Botany, Jiangsu Province and Chinese Academy of Sciences

Nanjing 210014, China

Tel: +86-25-84347086

E-mail: [yuanhaiyan416@163.com](mailto:yuanhaiyan416@163.com)

**
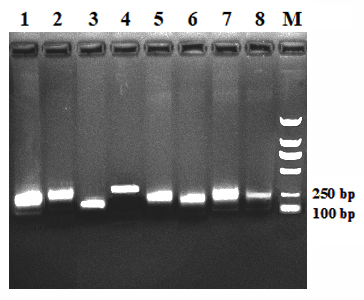
**

**Supplementary Figure S1.** Amplification results of 8 candidate genes with cDNA as templates. Lane1 *EF1α*, 2 *GAPDH*, 3 *ACT6*, 4 *UBQ*, 5 *UBC*, 6 *EF1β*, 7 *PGK*, 8 *TUB*. M: DL 2000 Plus DNA Marker (from up to low, 2000bp, 1000bp, 750bp, 500bp, 250bp, 100bp).

**
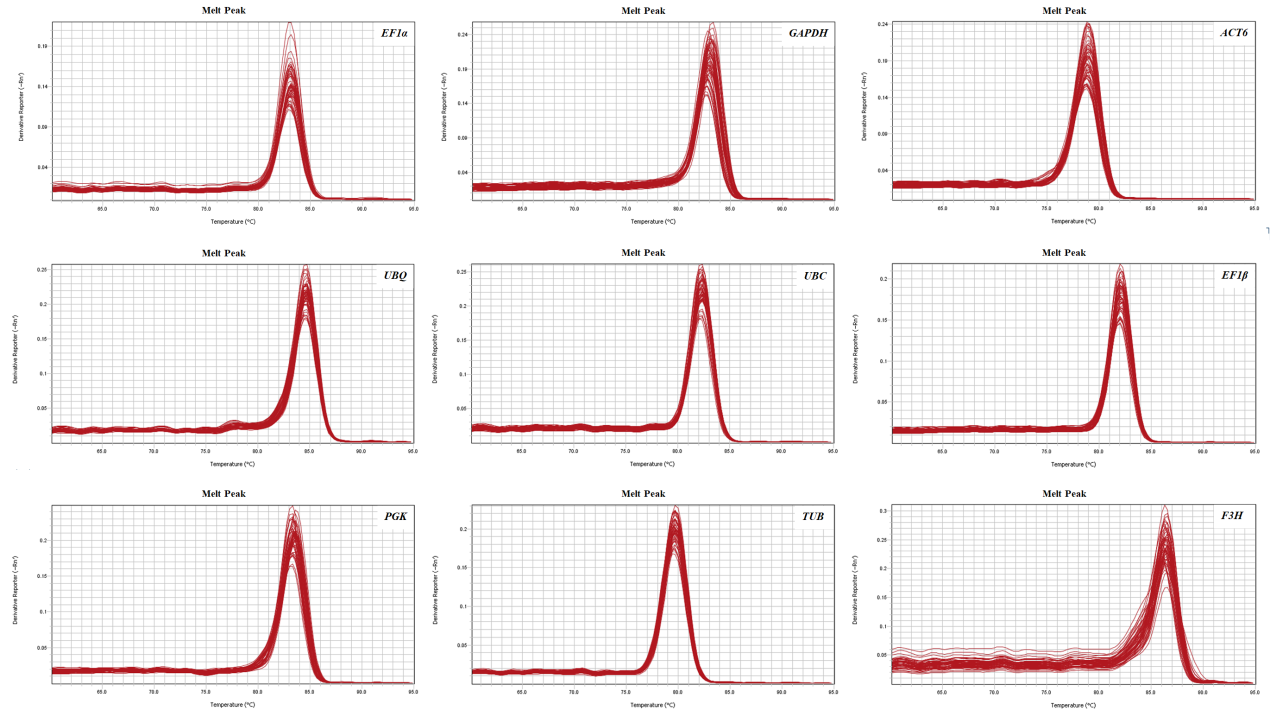
**

**Supplementary Figure S2.** Melting curves of 8 reference genes (*EF1α*, *GAPDH*, *ACT6*, *UBQ*, *UBC*, *EF1β*, *PGK* and *TUB*) showing single peak.

**Supplementary Table S1.** Raw Cq values of the 8 candidate genes in different flowering stages and different genotypes of *I. germanica* cultivars.

|  |  | *IgEF1ɑ* | *IgGAPDH* | *IgACT6* | *IgUBQ* | *IgUBC* | *IgEF1β* | *IgPGK* | *IgTUB* |
| --- | --- | --- | --- | --- | --- | --- | --- | --- | --- |
| ‘00246’ | FS1 | 21.01 | 25.36 | 23.02 | 23.02 | 19.98 | 21.60 | 22.10 | 21.41 |
|  |  | 20.69 | 25.48 | 22.85 | 23.01 | 20.05 | 21.60 | 22.15 | 21.45 |
|  |  | 20.66 | 25.31 | 22.82 | 22.99 | 19.98 | 21.57 | 22.16 | 21.52 |
|  |  | 19.51 | 23.84 | 22.34 | 24.01 | 20.45 | 22.08 | 20.12 | 20.73 |
|  |  | 19.23 | 23.89 | 22.59 | 24.06 | 20.41 | 22.11 | 20.76 | 20.82 |
|  |  | 19.92 | 23.77 | 22.67 | 24.09 | 20.39 | 21.86 | 20.24 | 20.79 |
|  |  | 21.24 | 25.82 | 23.59 | 21.92 | 19.69 | 21.25 | 23.72 | 22.18 |
|  |  | 21.83 | 26.03 | 23.52 | 21.97 | 19.71 | 21.08 | 24.15 | 22.10 |
|  |  | 21.73 | 26.12 | 22.68 | 21.99 | 19.68 | 21.17 | 23.82 | 22.09 |
|  | FS2 | 21.56 | 25.13 | 22.97 | 22.25 | 19.99 | 22.09 | 21.50 | 21.20 |
|  |  | 21.52 | 25.13 | 22.93 | 22.23 | 19.98 | 22.16 | 21.66 | 21.25 |
|  |  | 22.54 | 25.07 | 22.90 | 22.20 | 19.95 | 22.07 | 21.68 | 21.24 |
|  |  | 24.32 | 26.52 | 24.02 | 20.77 | 19.58 | 21.83 | 21.25 | 21.92 |
|  |  | 24.84 | 25.35 | 23.87 | 20.80 | 19.62 | 21.90 | 21.11 | 21.99 |
|  |  | 23.54 | 25.58 | 23.78 | 20.72 | 19.66 | 21.63 | 21.16 | 22.06 |
|  |  | 19.02 | 24.72 | 21.75 | 23.66 | 20.39 | 22.41 | 21.82 | 20.40 |
|  |  | 19.88 | 24.45 | 22.14 | 23.69 | 20.23 | 22.69 | 21.86 | 20.57 |
|  |  | 19.37 | 24.03 | 21.97 | 23.71 | 20.34 | 22.18 | 21.93 | 20.42 |
|  | FS3 | 22.34 | 24.23 | 23.96 | 20.78 | 20.31 | 22.31 | 22.65 | 21.84 |
|  |  | 22.30 | 23.45 | 24.20 | 20.84 | 20.34 | 22.28 | 22.84 | 21.82 |
|  |  | 22.24 | 23.93 | 24.10 | 20.85 | 20.25 | 22.30 | 22.69 | 21.74 |
|  |  | 23.48 | 25.16 | 22.63 | 21.59 | 19.93 | 21.45 | 21.93 | 22.61 |
|  |  | 22.83 | 24.94 | 22.91 | 21.63 | 19.82 | 21.58 | 21.94 | 22.55 |
|  |  | 23.54 | 24.77 | 23.02 | 21.68 | 19.99 | 21.51 | 21.90 | 22.79 |
|  |  | 20.77 | 25.91 | 24.81 | 19.93 | 20.71 | 23.03 | 23.08 | 20.88 |
|  |  | 21.39 | 25.75 | 25.22 | 19.99 | 20.69 | 23.16 | 23.68 | 20.93 |
|  |  | 21.73 | 25.82 | 24.91 | 20.12 | 20.65 | 23.08 | 23.55 | 21.01 |
|  | FS4 | 22.34 | 23.75 | 23.79 | 20.02 | 20.17 | 22.45 | 23.73 | 21.94 |
|  |  | 22.40 | 23.83 | 23.63 | 19.89 | 20.06 | 22.74 | 23.96 | 21.99 |
|  |  | 22.31 | 23.77 | 23.75 | 19.89 | 20.10 | 22.30 | 23.60 | 21.99 |
|  |  | 19.98 | 27.33 | 22.34 | 20.62 | 20.57 | 24.16 | 22.94 | 21.01 |
|  |  | 20.35 | 27.92 | 21.95 | 20.57 | 20.52 | 24.20 | 22.89 | 20.94 |
|  |  | 19.82 | 27.58 | 21.68 | 20.48 | 20.58 | 24.11 | 22.71 | 20.90 |
|  |  | 24.32 | 22.83 | 25.76 | 19.33 | 19.83 | 22.15 | 24.15 | 23.09 |
|  |  | 24.19 | 22.78 | 24.92 | 19.31 | 19.88 | 22.27 | 23.98 | 22.93 |
|  |  | 24.90 | 23.25 | 25.63 | 19.29 | 19.93 | 22.18 | 24.09 | 22.96 |
| ‘2010200’ | FS1 | 19.58 | 25.13 | 23.08 | 24.04 | 20.45 | 22.18 | 23.04 | 23.59 |
|  |  | 19.66 | 25.14 | 22.98 | 23.92 | 20.46 | 22.29 | 23.00 | 23.72 |
|  |  | 20.53 | 25.14 | 23.01 | 23.97 | 20.52 | 22.27 | 23.16 | 23.60 |
|  |  | 21.94 | 24.32 | 19.74 | 24.85 | 20.15 | 21.83 | 22.83 | 24.15 |
|  |  | 22.63 | 24.46 | 20.82 | 24.91 | 20.08 | 21.80 | 22.78 | 24.18 |
|  |  | 21.58 | 24.58 | 21.06 | 25.04 | 20.11 | 21.81 | 22.81 | 24.20 |
|  |  | 19.84 | 25.27 | 25.61 | 23.11 | 20.87 | 22.67 | 23.11 | 23.33 |
|  |  | 20.19 | 26.06 | 25.63 | 23.06 | 20.79 | 22.69 | 23.15 | 23.31 |
|  |  | 19.38 | 26.12 | 25.21 | 22.94 | 20.83 | 22.71 | 23.16 | 23.29 |
|  | FS2 | 20.65 | 25.94 | 22.53 | 23.33 | 19.92 | 22.28 | 23.18 | 22.24 |
|  |  | 20.60 | 25.87 | 22.43 | 23.27 | 19.98 | 22.25 | 23.10 | 22.25 |
|  |  | 20.62 | 26.04 | 22.45 | 23.25 | 20.04 | 22.20 | 23.13 | 22.24 |
|  |  | 21.54 | 25.92 | 23.81 | 22.79 | 19.83 | 21.32 | 22.75 | 21.59 |
|  |  | 22.28 | 25.81 | 22.96 | 22.93 | 19.89 | 21.39 | 22.40 | 21.63 |
|  |  | 21.61 | 24.89 | 23.73 | 22.86 | 19.80 | 21.35 | 22.97 | 21.67 |
|  |  | 20.01 | 26.12 | 21.01 | 23.71 | 20.13 | 22.74 | 23.13 | 22.92 |
|  |  | 19.43 | 26.28 | 21.83 | 23.66 | 20.07 | 22.78 | 24.09 | 22.85 |
|  |  | 19.35 | 26.72 | 21.46 | 23.72 | 20.09 | 22.91 | 23.15 | 22.80 |
|  | FS3 | 22.44 | 24.98 | 23.10 | 20.31 | 20.35 | 24.17 | 23.94 | 22.83 |
|  |  | 22.63 | 24.89 | 22.97 | 20.27 | 20.56 | 24.15 | 23.90 | 22.61 |
|  |  | 22.77 | 25.08 | 22.98 | 20.32 | 20.44 | 24.10 | 23.87 | 22.81 |
|  |  | 23.94 | 26.36 | 21.73 | 21.12 | 20.14 | 25.35 | 24.56 | 21.75 |
|  |  | 24.07 | 26.03 | 21.80 | 21.06 | 20.16 | 25.31 | 24.68 | 21.88 |
|  |  | 24.41 | 26.58 | 21.35 | 21.20 | 20.20 | 25.38 | 23.95 | 21.83 |
|  |  | 20.88 | 23.34 | 24.58 | 19.94 | 20.74 | 23.11 | 23.00 | 23.58 |
|  |  | 20.72 | 23.83 | 24.37 | 20.01 | 20.76 | 23.16 | 23.75 | 23.62 |
|  |  | 21.62 | 23.79 | 24.29 | 19.88 | 20.69 | 23.18 | 23.16 | 23.45 |
|  | FS4 | 24.93 | 27.62 | 21.88 | 22.49 | 21.69 | 25.72 | 26.16 | 22.84 |
|  |  | 24.92 | 27.49 | 21.85 | 22.58 | 21.66 | 25.73 | 26.37 | 22.89 |
|  |  | 24.82 | 27.60 | 21.88 | 22.60 | 21.82 | 25.57 | 26.35 | 22.92 |
|  |  | 25.92 | 26.82 | 20.58 | 23.34 | 20.33 | 24.83 | 26.87 | 21.85 |
|  |  | 25.97 | 26.47 | 20.30 | 23.31 | 20.24 | 24.94 | 27.01 | 21.79 |
|  |  | 25.71 | 25.92 | 21.43 | 23.39 | 20.19 | 24.89 | 26.96 | 21.72 |
|  |  | 24.18 | 29.25 | 23.19 | 21.80 | 23.11 | 26.27 | 25.67 | 23.86 |
|  |  | 23.93 | 28.93 | 23.32 | 21.89 | 23.19 | 26.30 | 26.05 | 23.98 |
|  |  | 23.60 | 28.43 | 22.36 | 21.64 | 23.25 | 26.35 | 25.20 | 24.07 |
| ‘Elizabeth’ | FS1 | 20.00 | 23.68 | 22.34 | 22.45 | 20.44 | 21.97 | 21.88 | 23.21 |
|  |  | 19.79 | 23.81 | 22.05 | 22.46 | 20.43 | 21.98 | 22.09 | 23.27 |
|  |  | 19.98 | 23.52 | 22.35 | 22.43 | 20.39 | 21.92 | 21.94 | 23.27 |
|  |  | 20.23 | 25.79 | 23.52 | 21.98 | 20.05 | 22.71 | 19.96 | 22.43 |
|  |  | 20.75 | 26.04 | 23.41 | 21.82 | 19.94 | 22.68 | 20.72 | 22.39 |
|  |  | 20.33 | 25.81 | 23.94 | 21.85 | 19.95 | 22.79 | 20.87 | 22.45 |
|  |  | 19.82 | 24.81 | 21.43 | 23.04 | 20.90 | 21.17 | 22.95 | 23.92 |
|  |  | 19.73 | 24.72 | 20.71 | 22.95 | 20.82 | 21.13 | 23.16 | 23.99 |
|  |  | 19.27 | 24.00 | 21.35 | 23.06 | 20.88 | 21.27 | 22.98 | 24.03 |
|  | **FS2** | 21.29 | 24.02 | 20.20 | 21.62 | 19.82 | 23.10 | 22.25 | 22.32 |
|  |  | 21.34 | 24.19 | 20.67 | 21.64 | 19.85 | 23.03 | 22.09 | 22.17 |
|  |  | 21.34 | 24.62 | 20.45 | 21.68 | 19.81 | 23.07 | 22.15 | 22.10 |
|  |  | 21.92 | 26.93 | 22.39 | 22.35 | 19.76 | 22.28 | 23.02 | 21.43 |
|  |  | 21.68 | 26.24 | 22.12 | 22.29 | 19.80 | 22.23 | 22.56 | 21.48 |
|  |  | 22.01 | 26.83 | 22.59 | 22.24 | 19.73 | 22.27 | 22.19 | 21.39 |
|  |  | 21.14 | 23.99 | 19.15 | 21.04 | 20.02 | 23.81 | 21.82 | 22.88 |
|  |  | 20.83 | 24.12 | 19.34 | 21.10 | 19.93 | 23.89 | 21.95 | 23.03 |
|  |  | 20.29 | 24.52 | 19.02 | 20.91 | 19.79 | 23.93 | 21.40 | 23.00 |
|  | FS3 | 21.93 | 24.83 | 22.33 | 19.99 | 20.12 | 24.51 | 23.92 | 23.02 |
|  |  | 22.07 | 24.79 | 22.29 | 19.98 | 20.14 | 24.60 | 23.89 | 23.04 |
|  |  | 22.18 | 24.86 | 22.30 | 19.97 | 20.16 | 24.56 | 23.98 | 23.07 |
|  |  | 21.67 | 24.27 | 22.58 | 19.37 | 20.00 | 23.76 | 24.13 | 22.30 |
|  |  | 21.87 | 23.83 | 22.16 | 19.42 | 19.93 | 23.79 | 24.27 | 22.36 |
|  |  | 21.64 | 23.56 | 22.56 | 19.46 | 19.99 | 23.72 | 24.18 | 22.41 |
|  |  | 22.35 | 25.65 | 22.81 | 20.41 | 20.36 | 25.36 | 23.61 | 23.72 |
|  |  | 21.93 | 25.94 | 22.13 | 20.55 | 20.29 | 25.38 | 23.73 | 23.84 |
|  |  | 22.19 | 25.78 | 21.62 | 20.68 | 20.27 | 25.38 | 23.68 | 23.80 |
|  | FS4 | 22.89 | 25.12 | 21.13 | 19.87 | 20.49 | 25.09 | 24.10 | 23.18 |
|  |  | 22.88 | 25.04 | 21.51 | 22.99 | 20.59 | 25.21 | 24.05 | 23.10 |
|  |  | 22.88 | 24.98 | 21.48 | 20.03 | 20.52 | 25.21 | 24.10 | 23.13 |
|  |  | 23.60 | 25.45 | 23.10 | 21.44 | 20.01 | 27.01 | 23.28 | 24.01 |
|  |  | 23.57 | 25.37 | 23.13 | 20.82 | 19.93 | 26.89 | 22.04 | 23.84 |
|  |  | 23.54 | 25.52 | 22.87 | 20.78 | 19.97 | 26.92 | 22.86 | 23.86 |
|  |  | 22.19 | 24.73 | 20.41 | 20.83 | 21.09 | 23.38 | 25.52 | 22.42 |
|  |  | 22.11 | 24.92 | 19.95 | 20.79 | 21.05 | 23.37 | 24.97 | 22.39 |
|  |  | 22.22 | 24.29 | 20.14 | 21.09 | 21.19 | 23.42 | 25.88 | 22.32 |

**Supplementary Table S2.** The traits of three different genotypes of *I. germanica* cultivars**.**

| Traits | ‘00246’ | ‘2010200’ | ‘Elizabeth’ |
| --- | --- | --- | --- |
| Flower color | Yellow (RHS 7D) | Yellow (RHS 4D) | Purple (RHS N82C) |
| Flowering period | 20 Mar.-10 Apr. | 05 Apr.–25 Apr | 20 Apr.–10 May |
| Number of flowers | 4-6 | 4-6 | 6-8 |
| Plant height (cm) | 55-65 | 75-80 | 80-90 |
